# Acoustic analysis of a single-cylinder diesel engine using magnetized biodiesel-diesel fuel blends

**DOI:** 10.1016/j.heliyon.2020.e05113

**Published:** 2020-10-01

**Authors:** Sadegh Samadi, Kobra Heidarbeigi

**Affiliations:** Mechanical Engineering of Biosystems Department, Ilam University, Ilam, Iran

**Keywords:** Energy, Fuel, Biodiesel, Magnetic, Sound pollution, Diesel engine, Signal processing

## Abstract

Fuels have important effects on the quality parameters of engines such as noise pollution and vibration. Diesel fuel are used in wide range of applications especially in compression ignition engines. The objective of the present research is evaluation of the noise emitted from a single-cylinder diesel engine using magnetized biodiesel-diesel fuel blends. In general, samples were provided with different percentages of the biodiesel-diesel blends as diesel (100, 95, 90, and 80%) and biodiesel (0, 5, 10, and 20%) by applying magnetic field (0, 5300 and 7000 G) to fuel line known as DxByMz. The measurements were done on a power tiller engine at a 10 cm distance from driver ear with three replications. The statistical approach in time domain and signal processing in frequency domain were applied for data analysis. The results of the variance analysis approved significant differences between the studied fuel blends and magnetic levels at 1% probability level. The highest and lowest average value of sound pressure level was corresponded to D100B0M0 and D80B20M5300, respectively. The results in frequency domain showed that the maximum sound pressure level values were in the frequency range of 31.5–200 Hz for all fuel blends and magnetic levels. The frequencies related to the maximum sound values varied by changing biodiesel percent and magnetic level.

## 1 Introduction

The role of self-propelled machines equipped by diesel engine significantly increased during the recent decades in different countries (Karthikeyan et al., 2020a). Diesel engines have vast applications in agriculture, transportation, construction and energy production due to high efficiency (Rangasamy et al., 2020). On the other hand, due to the development of the electronics industry and its application in engine, diesel engines are used in self-propelled machines more than before. There are two issues in this aspect, reduction of fossil fuel resources and environmental pollutions that causes to change the climate (Mirzajanzadeh et al., 2015; Karthikeyan et al., 2020b). The decrease of fossil fuel sources and increase of the prices of fossil fuels in the world caused to use of renewable energy sources in diesel engines as an alternative fuels. Moreover, environmental pollution emitted by ignition of fossil fuels more emphasized on the use of renewable energies (Zoldy, 2011; Ghobadian et al., 2009; Khoobbakht et al., 2019).

Biodiesel is prepared from vegetable oils, food crops and waste feedstock by transesterification (Khoobbakht et al., 2020a and b) and has approximately similar characteristics to diesel fuel especially in terms of cetane number (Costa and Piazzullo, 2018; Sindhanai Selvan et al., 2018).

Biodiesel is an oxygenated fuel (Ferreira et al., 2013; Szybist et al., 2007; Lin and Rong-Ji, 2009; Torkian Boldaji et al., 2011; Venkatesana and Nallusamy, 2019) that can be blended with diesel fuel. Therefore, a number of experimental studies have been presented to show the potential of biodiesel using in diesel engine (Rangasamy et al., 2020) and to evaluate the performance and exhaust emission of diesel engines fueling by proportions of biodiesel fuel (Abedin et al., 2014; Adaileh and AlQdah, 2012; Ahmed et al., 2014; Noorollahi et al., 2018; Najafi et al., 2007; Shirneshan et al., 2016). Jam et al. (2016) examined the impact of biodiesel fuel made from olive oil on the performance of a diesel engine. The results of their research showed that with increase in the biodiesel percentage blended in diesel fuel, the break mean effective pressure reduced but the specific fuel consumption increased. So that pure biodiesel compared to pure diesel caused to decrease break mean effective pressure by 13.4% and increase specific fuel consumption by 68.9%. Abbasi et al. (2018) analyzed the energy status of a diesel engine fueled by biodiesel derived from waste cooking oil. The research results showed that the maximum share of energy losses through the exhaust was belonged to 42% biodiesel (55.98%) and the minimum share was belonged to pure diesel as 46.48%. Khoobbakht et al. (2019) studied the effects of biodiesel on the performance indicators of a diesel engine and reported that the low level of biodiesel in the fuel could enhance the engine brake thermal efficiency in comparison with the pure diesel fuel or high level of biodiesel.

One of the important properties of diesel fuels is high noise and vibration. Sound and vibration resulting from the combustion in the engines have direct effects on the user's ears and body. So, noise pollution emitted from the engines fueled by fossil fuels is concerned in the global society (Beheshti and Ghandhari, 2015). One of the biodiesel advantages is less pollution in comparison with diesel fuels. Jahanbakhshi et al. (2017) examine the sound pollution of a grain combine harvester, model John Deere 1055I. Siavash et al. (2015) measured and analyzed sound pollution of a tractor engine fueled by different biodiesel-diesel blends (B0, B5, B10, B15, B20, B25, and B30) and they showed that biodiesel had effective effects on the sound pressure level of the studied engine.

Recently, various researches were conducted to magnetize the fuel before entering to the engine and also many patents have been recorded in this area in order to improve engine performance (Fatih and Gad, 2010; Bowker, 2000). The magnetic field orientates and polarizes the non-symmetrical (non-polarized) molecules of the fuels. The value of attraction forces between the hydrocarbon molecules of fuels under the influence of magnetic field is decreased and these molecules have more chance for reaction with oxygen molecules (Jain and Deshmukh, 2012). Based on the Van der Waals law, a strong link can be established between the hydrocarbon molecules in the magnetized fuels and oxygen molecules that guarantees an optimal combustion in the combustion chamber and reduce fuel consumption (Faris et al., 2012).

Available hydrogen in fuels as the main and the lightest element constitutes the main part of the hydrocarbon fuels, has a positive (proton) and a negative (electrons) part, i.e. it has bipolar torque (Govindasamy and Dhandapani, 2007a).

During recent years, the aim of the designers of internal combustion engines was to fight against the effects of the molecule clusters in hydrocarbon fuels to improve the combustion process (Guo et al., 1994). The appropriate magnetic field creates beneficial changes in the fuel structure and increases the reactive of fuels in the combustion process (Govindasamy and Dhandapani, 2007b). By applying proper magnetic field, H–C bonds in these hydrocarbon clusters are loosed and then crashed. In this status, these molecules have been separated from each other and move independently (Pera and Pines, 1987).

Darvishi et al. (2014) investigated on the exhaust emissions of a diesel engine under the effects of different magnetic field levels. The results of their research showed that magnetic field of 1000 G had the lowest amount of the CO emission whereas 3000 G had the minimum production of NOx and HC emissions. Patel et al. (2014) in a study on the performance and emissions of a four-stroke single-cylinder diesel engine, reported that it effects on fuel consumption, thermal efficiency, and exhaust emissions so that it decreased specific fuel consumption at full load by 8% and HC and NO_x_ by 30 and 27.7%, respectively. Also it reduced the CO emission.

Further, most of the previous researches on the effect of biodiesel fuel in diesel engines mainly focused on combustion characteristics, engine performance and exhaust emission. However, detailed sound analysis of engines under magnetized biodiesel-diesel fuel is missing in the literature, so it is essential to evaluate and convince the impact of these fuels on the engine standards in terms of noise pollution because engine acoustic emission should not exceed from certain threshold limit.

Considering the importance of fuel substitution (biodiesel) as well as the magnetic fuel and consequently engine combustion in one hand and the undesirable effects of engines sound pollution on human health, caused to conduct the present research. So, the novelty of the present research is using magnetized biodiesel-diesel fuel blends and studying the effects of this fuel on sound pollution of the diesel engine. The target machine of the present research was a single-cylinder diesel engine of agricultural tillers.

## 2 Materials and methods

The experiments were conducted in Mechanical Engineering of Biosystems Department, Ilam University, Ilam, Iran.

### 2.1 Diesel engine

In the present study, an agricultural power tiller engine manufactured by Mitsubishi Co. Japan, was used. Table 1 shows the technical specifications of the engine. The main factor in emitting of sound pollution in power tiller is the engine, therefore in this research, the engine as the part of the tiller was considered for evaluation of acoustic emission.

**Table 1:** The specification of the evaluated engine.

Specification	Value
Engine Model	ND75
Fuel type	Diesel
Power at 2200 rpm	7.5 hp
Number of cylinders	1
Engine cycle	4-Stroke
Air intake system	Naturally aspirated

### 2.2 Biodiesel fuel

In the present study, various blends of biodiesel and diesel fuels were prepared and used to evaluate the effects of biodiesel percentage on acoustic emission of the diesel engine. These blends were prepared with volumetric ratio of 0, 5, 10 and 20% biodiesel as B0, B5, B10, and B20. The B letter represents biodiesel fuel and the number after that specifies the percentage share in the blended fuel composition. In the present, biodiesel was purchased from biodiesel laboratory of Bioenergy Research Center, Tarbiat Modarres University, Iran, which was produced based on ASTMD 6751-09 standard. The specification of the used biodiesel was listed in Table 2. Also, D2 diesel fuel that used in this study was refined and produced in Iran according to ASTM D975 Standard.

**Table 2:** Biodiesel specifications.

Property	Unit	Value
Density	g.cm^−3^	0.88
Viscosity	mm^2^.s^−1^	4.73
Flash point	°C	176.00
Cloud point	°C	−1.00
Pour point	°C	−4.00
High heating value	Btu.gal^−1^	127.00

### 2.3 Data acquisition

The engine was fueled by different biodiesel-diesel blends with and without using magnetic field. The sound level at 10 cm distance from the driver ear was measured at 2200 engine rotational speed without load (Jahanbakhshi et al., 2016, 2017, 2020; Lashgari and Maleki, 2015, 2016).

In the present research, also six neodymium magnet grips 20 × 40 × 50 cm^3^ with maximum intensity of 32000 G were used for magnetizing the fuel blends. The two wooden boxes with inner dimension of 40 × 150 mm^2^ were used due to the high adhesion properties of the magnets. The two magnet distances, 5 and 10 cm, were considered to provide magnetic intensity of 5300 and 7000 G, respectively.

**Figure 1:**
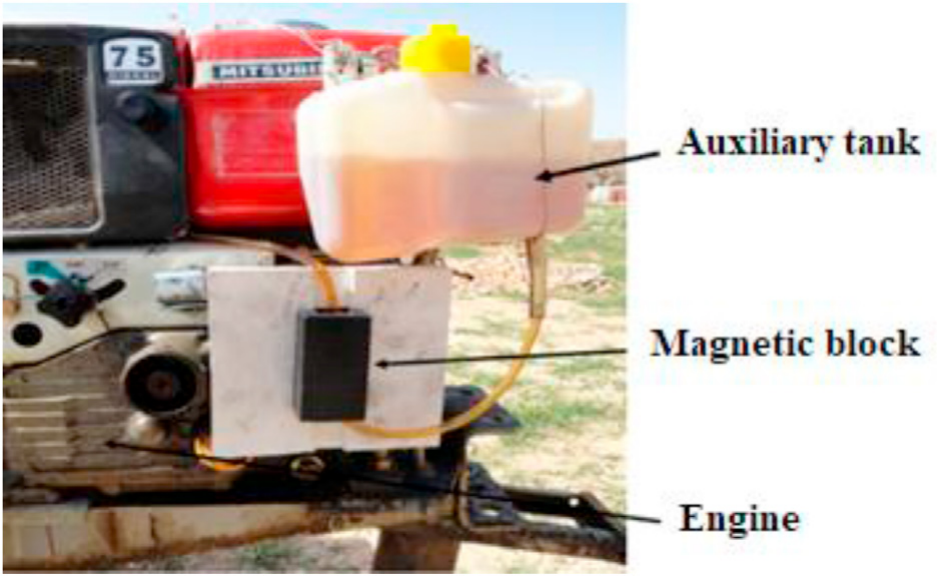
The placement of magnets along the fuel line.

The magnets must be installed in a proper place between fuel tank and engine (Figure 1). So the magnets were placed on the fuel line near to the engine (Habbo et al., 2011).

In order to measure the sound pressure level (SPL) of the studied engine, model SL 4013, Lutron, Taiwan, sound meter with electric capacitor microphone was used. The device had two outputs: alternating voltage (AC Output) and computer interface (RS232). The RS232 computer interface was used to measure and record the sound signals in the time domain. The microphone was connected to the sound meter device to sense the sound pressure and convert them to voltage data. To record the sound signals emitted from the tiller, the sensor was connected to data logger and the signals were transmitted to a personal computer using a RS232 cable.

Environmental characteristics and exposer of operator are important to proper measure and evaluate sound signals. The location of the experiments was selected based on the international organization standards and the Association of Automotive Engineering. It must be an open, flat and free of ash or snow and far from big reflectors such as buildings and trees (Anonymous, 1985; Anonymous, 1996). Also the tests must be done with environmental conditions without atmospheric precipitation and wind speed of less than 5 m.s^−1^.

In the present research, the environment conditions such as wind speed, sound and ambient air temperatures were measured so that they were 1.8 ± 1 m.s^−1^, 46 ± 1 dB, and 12 ± 1 °C, respectively. For measuring wind speed and ambient temperature an anemometer, model AM-4206, was used.

**Figure 2:**
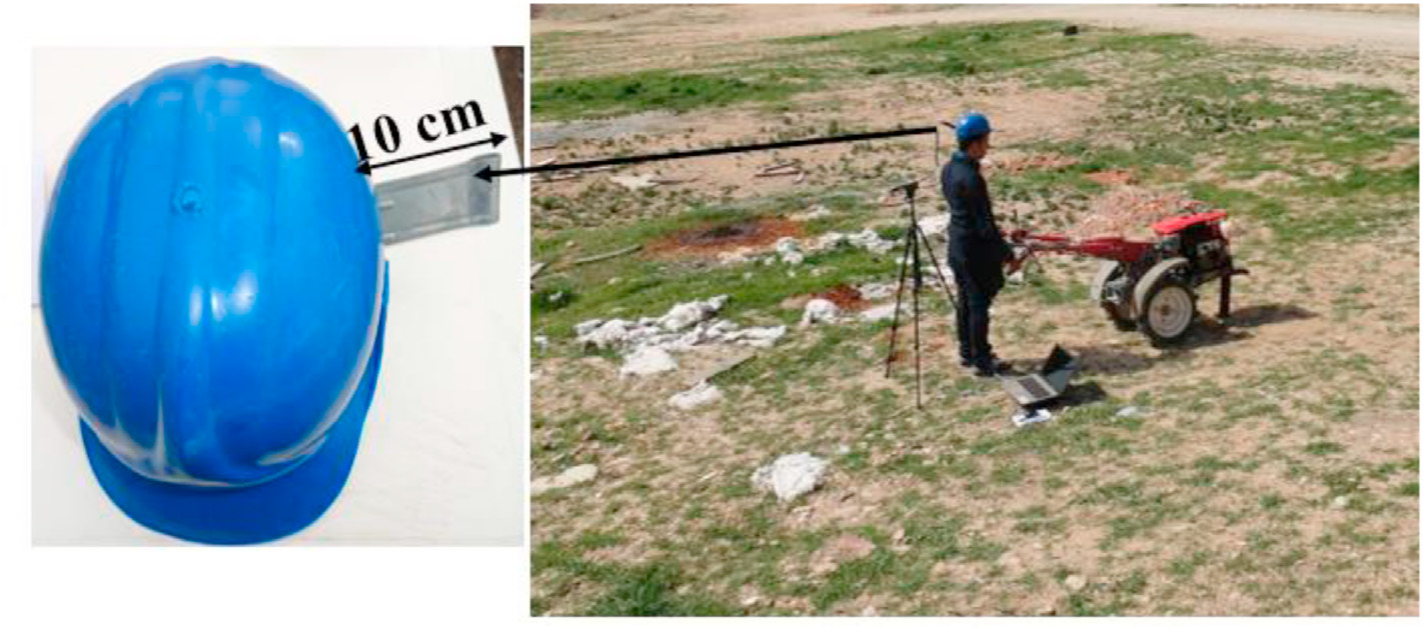
Measuring the sound level of the tiller in the test station.

After calibration the measuring device, the sound level (A network) was measured considering the slow response speed of the device. The microphone was installed on a clamp attached to the driver helmet to measure the sound levels at a distance of 10 cm from driver's ears (Figure 2).

To record the sound data, the Lutron 801 Software, model SW-U801-WIN, was used. In each test, the sound data was recorded for 2 min with 2 s time intervals of each signal.

The uncertainty and accuracies of the used instruments in the present study were showed in Table 3. Due to these uncertainties, the error in the measured parameters can be calculated for each result. Accordingly, error analysis was performed using the Gaussian Distribution Method with 95% confidence level (Eq. 1)(Table 4).

(1) $$Percentage\;of\;analysis\;uncertainty=\sqrt{{\left(speed\right)}^{2}+{\left(SPL\right)}^{2}+{\left(Fuelmeasuring\right)}^{2}+{\left(Gauss\right)}^{2}+{\left(AirFlow\right)}^{2}+{\left(Temprature\right)}^{2}}=\pm \sqrt{{\left(0.05\right)}^{2}+{\left(0.1\right)}^{2}+{\left(1\right)}^{2}+{\left(1\right)}^{2}+{\left(2\right)}^{2}+{\left(2\right)}^{2}}=\pm 3.16\%\;$$

**Table 3:** Uncertainty of different instruments.

Measured parameter	Accuracy	Uncertainty (%)
Speed (rpm)	±1	0.05
Sound pressure meter (dB)	±2	0.1
Fuel flow rate (cc)	±0.1	1
Gauss meter (G)	±0.1	1
Air speed (m.s^−1^)	±0.01	2
Temperature (°C)	±0.1	2

**Table 4:** Experimental conditions to measure engine sound.

Variables level	1	2	3	4
Fuel blends (% biodiesel)	0	5	10	20
Magnetic field (G)	0	5300	7000	-

### 2.4 Acoustic analysis

In the present study, statistical analysis of the obtained data in time domain was performed as factorial experiment based on completely randomized design using SAS 9.1 Software. The main factors in the present investigation were fuel with four levels (B0, B5, B10 and B20) and magnetic field at three levels (M0, M5300 and M7000 G). Each treatment was tested at three replications and totally 36 (=4 × 3 × 3) tests were conducted .

**Figure 3:**
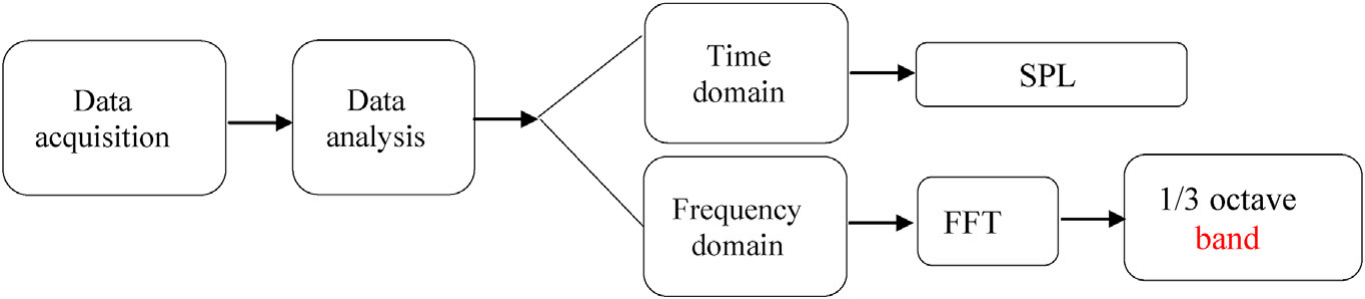
Procedure of acoustic signals analysis.

The measured attributes were sound signals. Experiments were performed at constant engine speed and in three replications (Table 3). Time domain analysis of signals shows the general trend of data changes due to the variables, but it is necessary to convert the signals frequency domain to obtain further information. Often the time response function does not provide much useful information, while the frequency response represents one or more separate frequencies where their energy is concentrated (Ahmadian et al., 2013). There are two main reasons for obtaining frequency content, 1) the response of human organs and mechanical systems depends on frequency and 2) the physical processes of propagation, transmission, and energy content of acoustic signals depend on their frequency. So it is necessary to convert the signals from the time to the frequency domain to achieve the narrow band spectrum of sound. Figure 3 shows the procedure of analyzing of sound signals. To convert the data from time domain to frequency domain, Fast Fourier Transform (FFT) was used in MATLAB R2010b Software environment.

## 3 Results and discussion

Sound pressure levels for all fuel blends and magnetic field intensities were obtained. Statistical analysis was used to evaluate the significance of the difference between the two-way interaction and main effects. The results of the variance analysis of the sound level at the driver's ear in 10 cm distance have been presented in Table 5. The results of the statistical analysis cleared that the effects of main variables, magnetism and biodiesel, on the engine noise were significant at 1% probability level. Also the interaction of magnetism with biodiesel on engine sound was significant at 1% probability level. These results indicated that there are important differences in the magnitude of SPL in different fuel blends and magnetic field intensity. According to significant effect of magnetism and biodiesel interaction on sound, the mean compression of the treatments was done by Duncan's Multiple Range Test (Table 6).

**Table 5:** The variance analysis of the tiller sound data.

SOV	DOF	SS	MS	F
Magnetism	2	18.12	9.06	43.39∗
Biodiesel	3	52.98	17.66	84.59∗
Magnetism×Biodiesel	6	16.63	2.77	13.28∗
Error	24	5.01	0.21	-
Total	35	92.74	-	-

∗ Significant at 1% probability level.

**Table 6:** Mean compression between the studied treatments.

Magnetic intensity	Biodiesel			
	B0∗	B5	B10	B20
M0	80.80^a^∗∗	78.82^c^	79.56^bc^	77.96^d^
M5300	79.75^b^	79.03^bc^	76.29^f^	75.59^f^
M7000	79.17^bc^	79.15^bc^	76.25^f^	77.18^e^

∗ B indicates biodiesel and M indicates magnetic intensity.

∗∗ Non-common letters show significant at 1% probability level.

As seen in Table 6, B0, B5, B10, and B20 had significant differences without magnetic field. There is no significant difference between B0 and B5 and between B10 and B20 when using magnetic intensity of 5300 G (M5300). For M7000, the difference between B0 and B5 was not significant but significant difference was observed between B10 and B20.

From Table 5 can be concluded that magnetic intensity of 7000 G (M7000) had lower effect on reduction of power tiller SPL than 5300 G (M5300). Also the effect of B20 fuel blend on power tiller SPL was higher than the other fuel blends so that the lowest SPL was emitted when using B20 fuel with 5300 G magnetic intensity (D80B20M5300).

### 3.1 Effect of biodiesel on sound emission

**Figure 4:**
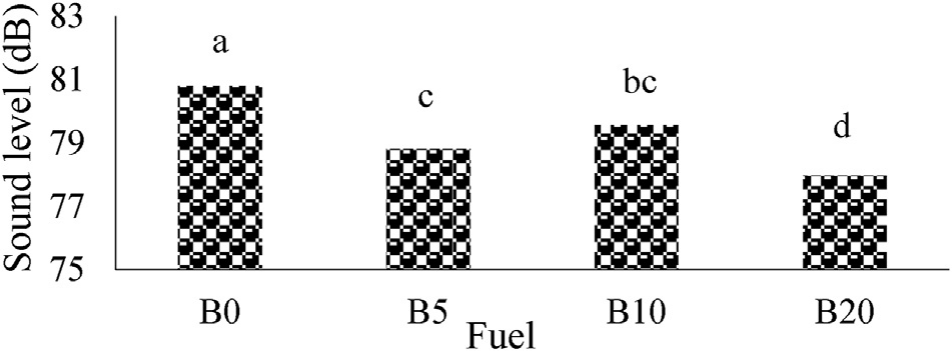
The average of sound pressure level for M0.

The average sound pressure level for different fuel blends without magnetism (M0) was showed in Figure 4. As shown in Figure 4, by increasing the amount of biodiesel percentage in the blends, the tiller SPL significantly changed at 1% probability level. The maximum average of SPL belonged to B0 with value of 80.80 dB that can be due to combustion knock and the minimum value (77.96 dB) was observed for B20 (D80B20M0). The same results have been reported by Siavash et al. (2015).

**Figure 5:**
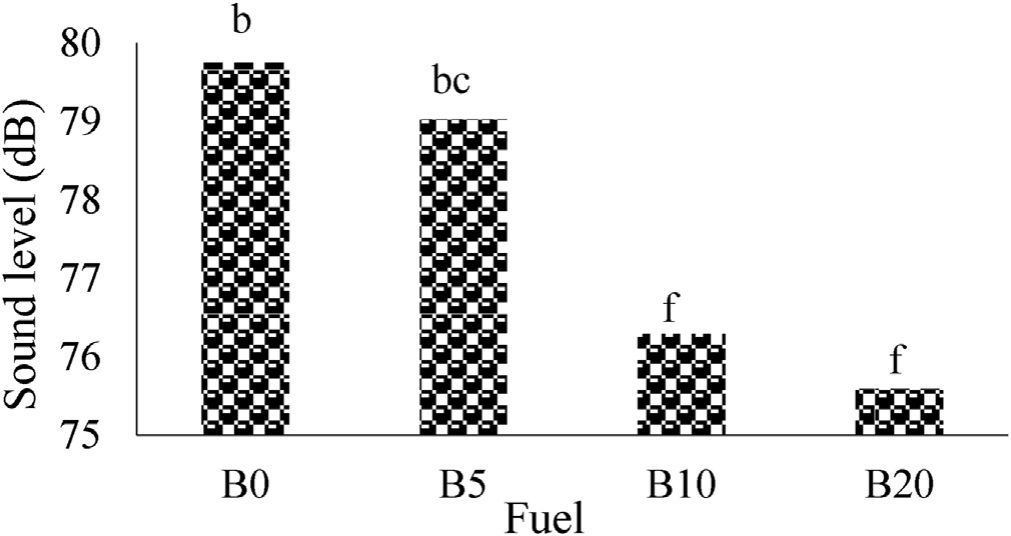
The average of sound pressure level for M5300.

The results of comparing the average of SPL corresponding to the studied fuel blends with using 5300 G magnetic field intensity (M5300) were showed in Figure 5. The results show that the highest SPL corresponds to B0 with sound level of 79.75 dB and B20 fuel blend has the lowest sound value with average of 75.59 dB. As indicated, SPL decreases by increasing of biodiesel percentage at M5300.

**Figure 6:**
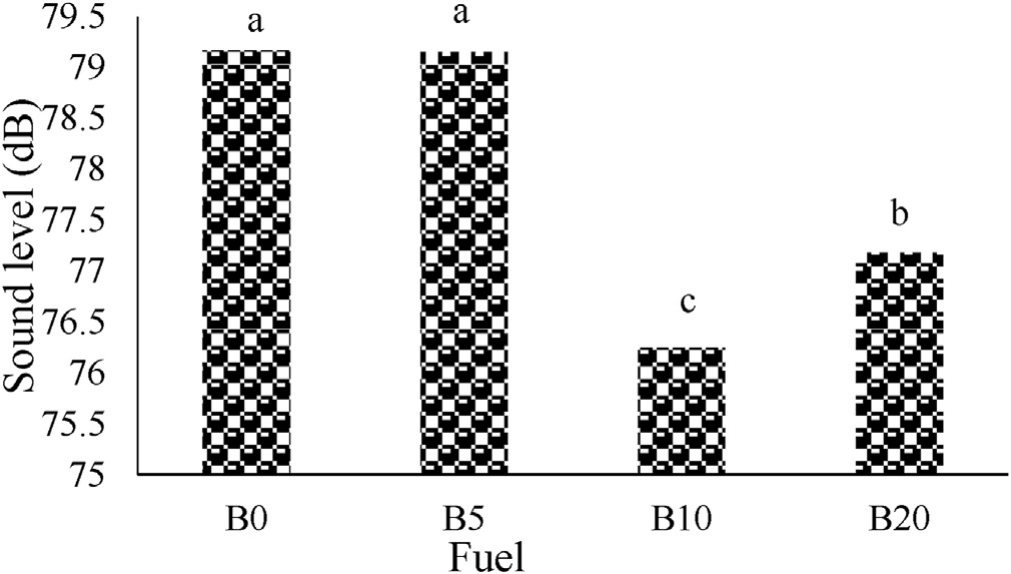
The average of sound pressure level for M7000.

Figure 6 shows the average of SPL with 7000 G magnetic intensity. The results don't show significant difference between B0 and B5 and these fuel blends had highest SPL with value of 79.17 and 79.15 dB, respectively. The sound average corresponding to B10 and B20 had significant difference at 1% probability level and the lowest sound value were related to B10 with value of 76.25 dB.

### 3.2 Frequency analysis

**Figure 7:**
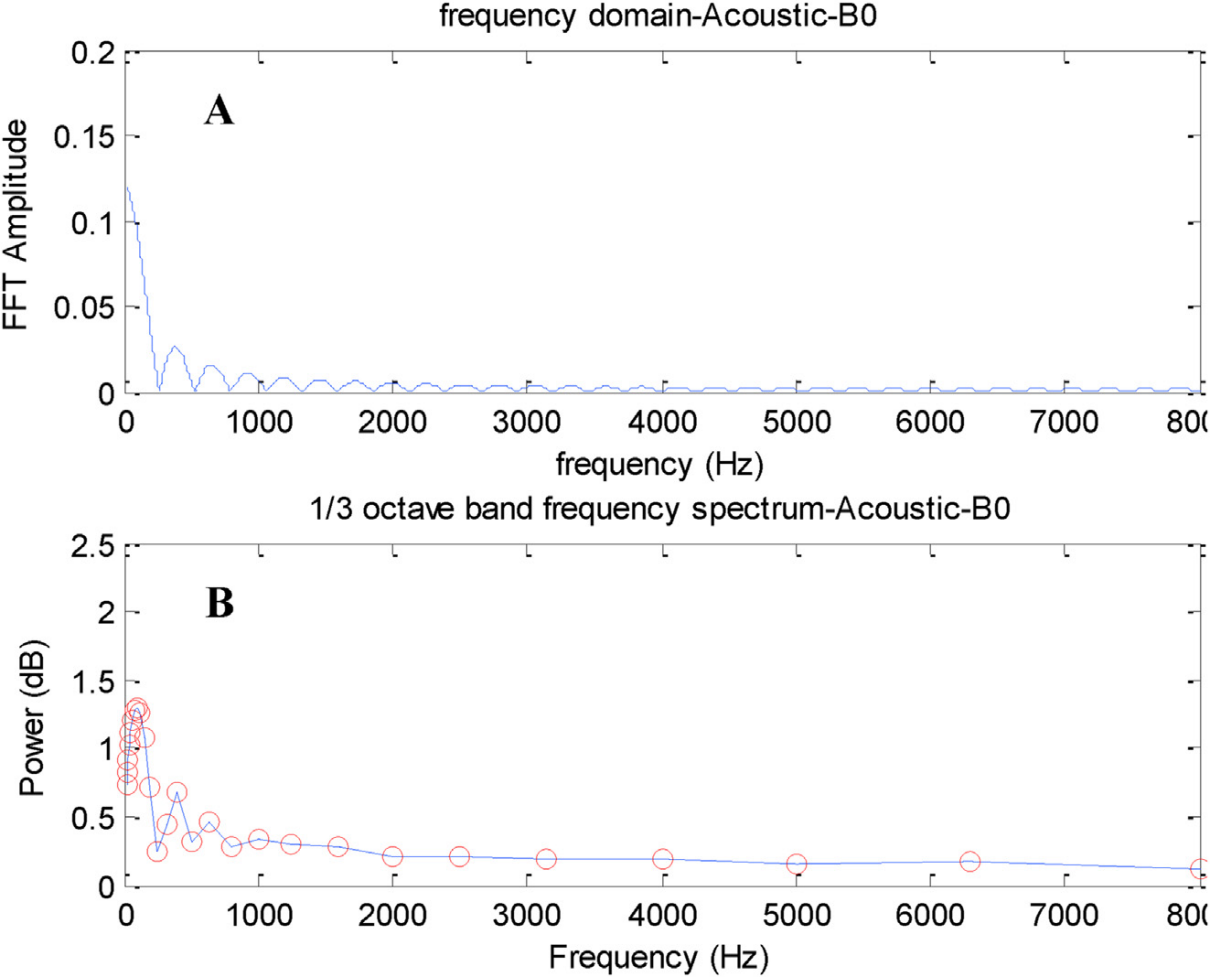
The sound data at (A) frequency domain and (B) 1/3 octave band for B0.

Figure 7 shows the signals from the frequently domain and 1/3 octave band of sound pressure level for B0 fuel. The diagrams were the output of MATLAB Software. The results of the data from the software output were transferred to Excel Software and then some diagrams were plotted.

**Figure 8:**
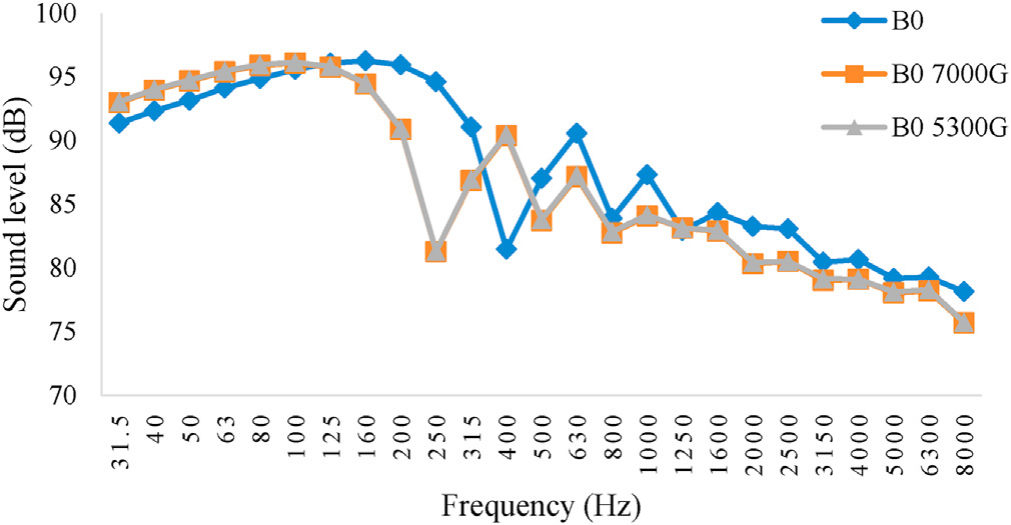
Frequency analysis of 1/3 octave band of sound level for B0.

**Figure 9:**
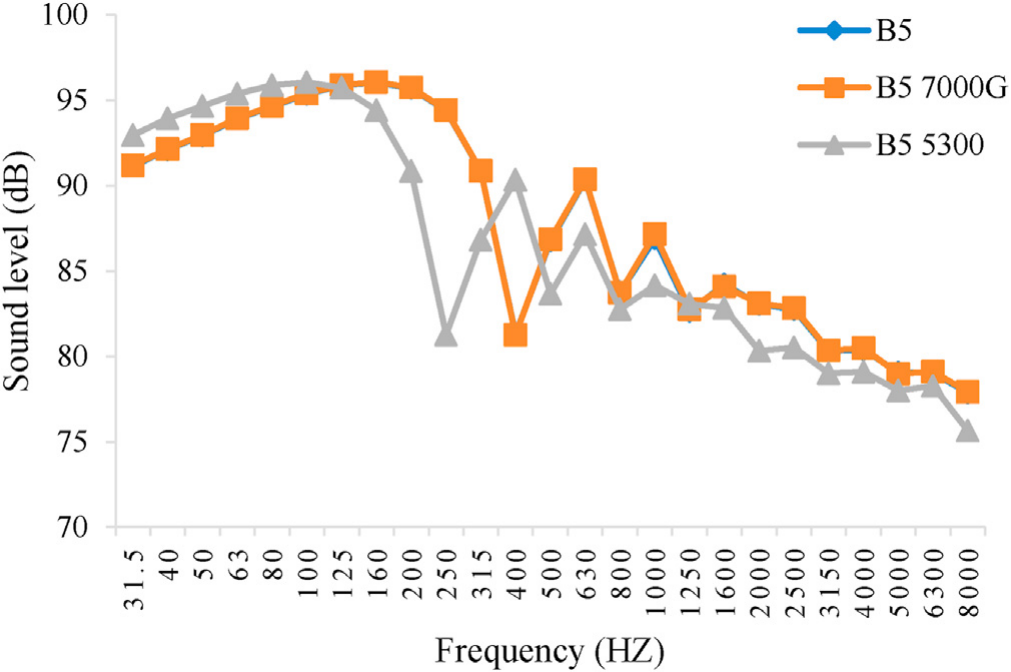
Frequency analysis of 1/3 octave band of sound level for B5.

**Figure 10:**
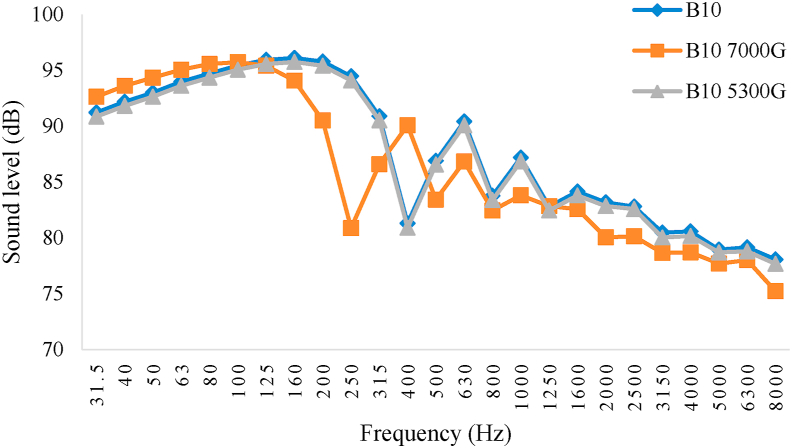
Frequency analysis of 1/3 octave band of sound level for B10.

**Figure 11:**
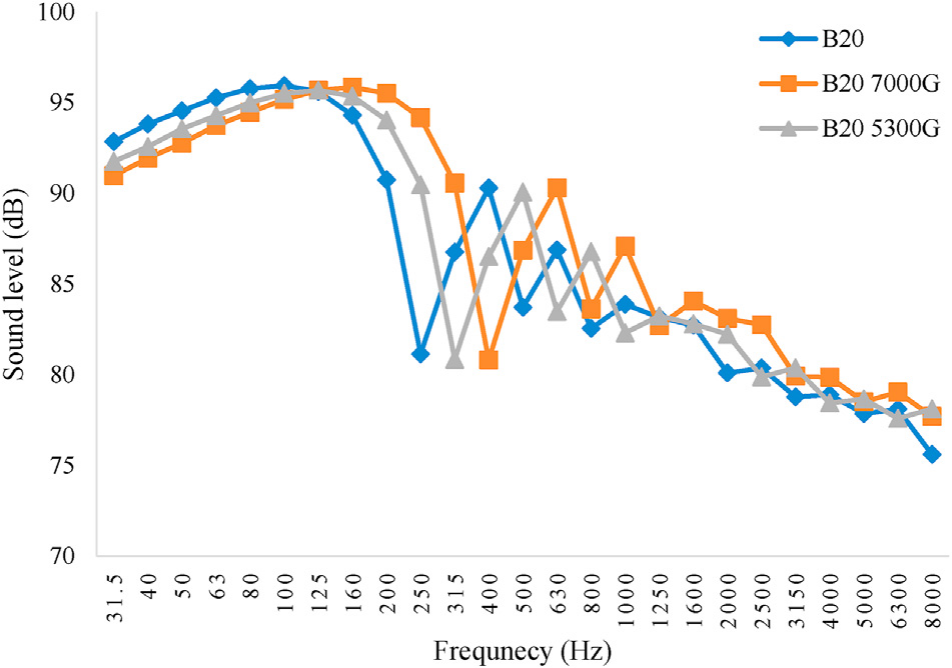
Frequency analysis of 1/3 octave band of sound level for B20.

Figures 8, 9, 10, and 11 show the diagram of frequency analysis of sound data for fuel blends of B0–B20. The diagrams show that the maximum sound pressure values for all fuel blends and magnetic intensities are in the range of 31.5–200 Hz. This frequency range is rooted to combustion process and firing quality. For the tested engine at this experiment, it was observed that SPL of engine in all fuel blends reduced in this frequency range by applying magnetic field. Also the frequencies corresponding to the maximum SPLs changed by changing biodiesel percentage and magnetic intensity. These changes were more observed for frequencies from 250 to 800 Hz that resulted from exhaust structure. So that the lowest amount of sound level belonged to B0 with M5300 and M7000, B5 and B10 with M5300 and also B20 with M0. Generally, the M5300 had the greatest impact on reduction of sound level.

The effective range that matches with human hearing system are frequencies between 300 and 3000 Hz which combustion and exhaust (muffler) design are the source of this range. For the used engine at this experiment, using magnetic field, it was observed that SPL of engine reduced for all fuel blends in this frequency range. It should be noted that magnetism breaks down the chain link between the hydrocarbons that consequently causes to reduce the density and surface tension of the fuel therefore more atomize the particles entering the combustion chamber. This causes to increase the fuel evaporation and have better combination of fuel with oxygen and then fuel oxidation. The consequences of this phenomenon are happening more complete combustion and reducing sound emission level (Darvishi et al., 2014).

## 4 Conclusion

In the present research after data acquisition and analysis there was specified that both main factors, biodiesel fuel and magnetic field intensity, and the interaction between those, had significant effect on the sound pressure level of the studied power tiller in the present research at 1% probability level. According to the Duncan Multiple Range Test, there were significant differences between B0, B5, B10 and B20 with and without using magnetic field on the fuel line. The maximum sound level was corresponded to pure diesel (B0) with average value of 80.80 dB without using magnetic field (M0) that can be due to combustion knock and the minimum SPL value was belonged to B20 and B10 with magnetic field intensity of 5300 G (M5300) and B10 with magnetic field intensity of 7000 G (M7000) as 75.59, 76.29, and 76.25 dB, respectively. Frequency analysis of 1/3 octave band spectrum showed that the maximum sound pressure level values for all studied fuel blends and magnetic intensities were in the most overcome frequencies of 31.5–200 Hz. Also the results showed that the frequencies corresponding to the maximum sound values changed by changing the biodiesel percentage and magnetic field intensity so that the lowest sound levels of B0 were related to 5300 and 7000 G field intensity, for B5 and B10 related to 5300 G and B20 related to 0 G magnetic field intensity. In addition, there was observed that the highest and lowest average value of SPL was corresponded to D100B0M0 and D80B20M5300, respectively. Also, the effective range that matches with human hearing system are frequencies between 300 and 3000 Hz which combustion and exhaust (muffler) design are the source of this range. For the evaluated engine, it was observed that SPL of engine for all fuel blends reduced in this frequency range by applying magnetic field. This result indicated that magnetic field significantly influence in smooth performance of the engine and reducing the engine SPL.

## Declarations

### Author contribution statement

Sadegh Samadi: Performed the experiments; Wrote the paper.

Kobra Heidarbeigi: Conceived and designed the experiments; Analyzed and interpreted the data; Contributed reagents, materials, analysis tools or data; Wrote the paper.

### Funding statement

This research did not receive any specific grant from funding agencies in the public, commercial, or not-for-profit sectors.

### Competing interest statement

The authors declare no conflict of interest.

### Additional information

No additional information is available for this paper.
